# Urban–rural differences in cardiovascular health among young adults in the Turkestan region of Kazakhstan

**DOI:** 10.3389/fpubh.2026.1873662

**Published:** 2026-08-19

**Authors:** Khandulla Nakipov, Balnur Omarova, Ainash Oshibayeva, Gulnaz Nuskabayeva, Raushan Kuandykova, Seitkhan Djoshibaev, Meiramgul Tundybayeva, Lyazat Baglanova

**Affiliations:** 1 Khoja Ahmet Yassawi International Kazakh Turkish University, Turkistan, Kazakhstan; 2 Research Clinical Center of Cardio Surgery and Transplantology, Khoja Ahmet Yassawi International Kazakh Turkish University, Turkistan, Kazakhstan; 3 Asfendiyarov Kazakh National Medical University, Almaty, Kazakhstan; 4 Kazakh-Russian Medical University, Almaty, Kazakhstan

**Keywords:** cardiovascular health, gender, Kazakhstan, life's essential 8, rural-urban

## Abstract

**Background:**

Cardiovascular disease (CVD) remains a major global health challenge, and early action among young populations is critical. The aim of this study was to assess cardiovascular health among young adults aged 18–44 years in the Turkestan region using the American Heart Association Life's Essential 8 (LE8) framework and to explore health care workers' perspectives on determinants of cardiovascular health and primary health care (PHC) strategies for prevention.

**Methods:**

A mixed-methods study design was used. In the quantitative component, cardiovascular health was assessed among adults aged 18–44 years attending urban and rural PHC facilities using an adapted LE8 approach. The overall LE8 score was calculated as the arithmetic mean of eight standardized component scores (0–100). Continuous and categorical variables were analyzed using independent-samples *t*-tests and chi-square (χ^2^) tests, respectively. In the qualitative component, semi-structured interviews were conducted with PHC nurses involved in preventive and screening programs. Interviews continued until thematic saturation was achieved and were analyzed using conventional thematic content analysis.

**Results:**

A total of 843 young adults participated in the study (urban: 432; rural: 411), including 289 males (34.3%) and 554 females (65.7%). Most participants had intermediate cardiovascular health (67.6%), while 28.9% had high and 3.4% had poor cardiovascular health according to the LE8 framework. Rural participants reported a healthier diet, higher physical activity, and longer sleep duration but also had substantially higher smoking prevalence and obesity. Urban participants achieved higher overall LE8 scores, largely due to lower tobacco exposure. Females, particularly in urban areas, had more favorable cardiovascular health profiles, with better smoking and sleep scores and higher overall LE8 scores than males. Interviews with seven PHC nurses indicated that cardiovascular risk factors increased after the age of 40, were more common among men, and were influenced by cultural, behavioral, and economic factors. Nurses emphasized the need to strengthen workplace- and community-based cardiovascular prevention programs.

**Conclusion:**

Most young adults in the Turkestan region demonstrated moderate cardiovascular health; however, modifiable cardiovascular risk factors remain common. These findings support the implementation of gender-specific and region-specific cardiovascular prevention strategies, culturally tailored lifestyle interventions, tobacco-control measures, and earlier cardiovascular screening within PHC settings.

## Introduction

Cardiovascular disease (CVD) remains a major global health challenge and a leading cause of mortality, morbidity, and healthcare expenditure. Despite substantial progress in reducing smoking prevalence, stroke mortality, and cardiovascular deaths over recent decades, the global burden of CVD is rising again due to worsening behavioral risk factors, population aging, persistent health inequities, and the long-term effects of the COVID-19 pandemic. In the United States alone, approximately 127.9 million adults live with CVD, while more than 71% are overweight or obese (1). Worldwide projections further suggest a substantial increase in CVD burden by 2050, with ischemic heart disease and elevated systolic blood pressure remaining among the leading contributors to mortality and disability (2). In Europe, CVD accounts for more than 3 million deaths annually, with significant disparities observed between countries, socioeconomic groups, and age categories (3). Adults younger than 70 years contribute more than 60 million potential years of life lost annually, highlighting the urgent need for strengthened prevention, surveillance, and targeted public health interventions (4).

Growing evidence demonstrates that cardiovascular risk begins early in life. Lifestyle and clinical factors during childhood, adolescence, and young adulthood strongly predict cardiovascular events later in adulthood (5). Longitudinal studies indicate that early-life risk factors, particularly obesity and elevated low-density lipoprotein cholesterol, contribute both directly and indirectly to future CVD, and these adverse effects may not be fully reversed even when health behaviors improve later in life (6). These findings emphasize the importance of early and proactive prevention strategies, particularly among young populations.

To support population-based prevention efforts, the American Heart Association developed the Life's Essential 8 (LE8) framework, a comprehensive cardiovascular health metric that includes eight modifiable health behaviors and factors: diet, physical activity, nicotine exposure, sleep, body mass index, total cholesterol, blood glucose, and blood pressure (7). Higher LE8 scores are consistently associated with lower risks of cardiovascular disease, stroke, mortality, cognitive decline, and other chronic conditions, making this framework a valuable tool for cardiovascular health monitoring and preventive interventions.

Evidence from Central Asia and other post-Soviet countries indicates that cardiovascular disease remains a leading cause of premature mortality despite ongoing health system reforms. Previous studies have reported a high prevalence of modifiable cardiovascular risk factors, including hypertension, obesity, tobacco use, and unhealthy dietary patterns, while population-based assessments of cardiovascular health using comprehensive metrics such as LE8 remain scarce (8). Consequently, evidence on cardiovascular health among young adults in this region is limited, highlighting an important knowledge gap.

Kazakhstan has strengthened primary health care (PHC) and expanded national cardiovascular screening and prevention programs delivered through PHC facilities. These programs provide an appropriate setting for assessing cardiovascular health among young adults (9).

Despite these improvements, life expectancy in Kazakhstan remains substantially lower than in many European countries, and a pronounced gender gap persists, with females living significantly longer than males (10). Several health system barriers continue to limit the effectiveness of PHC prevention programs, including limited workforce capacity and insufficient training in population-based prevention and patient counseling. Historically, the post-Soviet educational model did not prepare advanced practice nurses capable of independently managing cardiovascular risk factor modification, nor did it include structured training for clinical psychologists. Although reforms have been introduced during the past two decades, these gaps continue to challenge effective preventive care delivery.

Recent national data further demonstrate declining avoidable mortality from cancer but persistently high and increasing mortality from cardiovascular diseases, emphasizing the urgent need to strengthen CVD prevention efforts in Kazakhstan (11). Understanding cardiovascular health in younger populations is particularly important, as early-life risk factors contribute substantially to future disease burden and premature mortality. Early prevention during young adulthood therefore offers a critical opportunity to reduce long-term cardiovascular risk.

This study was conducted in the Turkestan region of southern Kazakhstan and included participants from both urban and rural primary health care centers to capture potential regional differences in cardiovascular health and behavioral risk factors. The aim of this study was to assess cardiovascular health among young adults aged 18–44 years using the LE8 framework and to explore healthcare workers' perspectives on determinants of cardiovascular health and strategies to strengthen cardiovascular prevention within primary health care.

## Methods

We used a mixed-methods approach. In the first step, we identified cardiovascular health components based on the American Heart Association (AHA) LE8 methodology (7). Data collection was conducted among individuals aged 18–44 years attending PHC facilities for non-urgent reasons in both urban and rural areas of the Turkestan region of Kazakhstan. Five PHC centers were selected purposively to represent both urban and rural settings within the Turkestan region. Selection was based on geographic location, population coverage, and willingness to participate in the study. These centers serve populations with differing sociodemographic characteristics, allowing inclusion of both urban and rural residents.

In Kazakhstan, PHC facilities maintain population registers and are responsible for implementing preventive examinations and national screening programs. Participants were recruited from the registered catchment population of the participating PHC centers through invitations issued by PHC nurses rather than exclusively from patients seeking medical care. Recruitment aimed to include adults across different age groups, while attendance at the PHC facility was necessary to obtain standardized clinical measurements, including anthropometry, blood pressure, and blood glucose assessment (12).

Trained nurses administered a structured questionnaire and performed clinical measurements, including blood pressure and capillary blood glucose assessment. Blood glucose was classified according to the LE8 scoring criteria using fasting blood glucose and/or HbA1c values. Because the Kazakhstan national cardiovascular screening program routinely measures total cholesterol rather than non-HDL cholesterol, the lipid component was based on total cholesterol values. This represents a deviation from the original AHA LE8 framework and reflects routine clinical practice in Kazakhstan, where total cholesterol is measured during national cardiovascular screening. Anthropometric measurements were also obtained according to standard clinical procedures. Alcohol consumption was not assessed because it is not included in the LE8 framework, although it represents an important cardiovascular risk factor in Kazakhstan. All information was initially recorded using paper-based forms and subsequently entered into Microsoft Excel for data management and analysis.

Cardiovascular health was assessed using an adapted version of the LE8 framework. Because several study variables were collected in categorical form, the original data were transformed into standardized component scores ranging from 0 to 100. The overall LE8 score was calculated as the arithmetic mean of the eight component scores, with higher scores indicating more favorable cardiovascular health. Dietary intake was assessed using a DASH-based dietary questionnaire and converted to a 0–100 diet score. Physical activity, smoking status, sleep duration, body mass index, total cholesterol, blood glucose, and blood pressure were also converted into standardized 0–100 component scores.

*Statistical analysis* was performed using IBM SPSS 23. Continuous variables were presented as means and standard deviations, and categorical variables as frequencies and percentages. Gender-based comparisons were conducted using the independent samples *t*-test for continuous variables and the chi-square (χ^2^) test for categorical variables. A *p*-value < 0.05 was considered statistically significant.

### Sample size

According to 2024 national demographic data, the population of Turkestan region aged 18–44 years is approximately 1,000,000 people. The minimum required sample size for a cross-sectional study was calculated using the standard formula:


$$\begin{array}{l} n=deff\;\times \;\frac{\left[N\;\cdot \;pˆ\left(1\;-\;pˆ\right)\right]}{\left(\left(N\;-\;1\frac{d^{2}}{z^{2}}\right)\;\right)\;+\;pˆ\left(1\;-\;pˆ\right)}\; \end{array}$$ (1)

Where:

N = 1,000,000 (population size)

Deff = 1 (design effect, random sampling)

*P* = 0.5 (assumed proportion – maximum variability)

*d* = 0.05 (margin of error)

*z* = 1.96 (95% confidence level)

Calculated minimum sample size: ≈ 384 participants.

The final study sample included 843 participants, exceeding the minimum required sample size and therefore considered statistically adequate for population-level inference.

In the second stage of the study, a qualitative descriptive approach was applied using semi-structured in-depth interviews. The interviews were conducted among nurses responsible for screening and preventive programs within PHC settings, implemented according to national protocols and regulatory documents of Kazakhstan. Participant characteristics are presented in Supplementary material 1. According to the national PHC Standard of Kazakhstan, nurses have a central role in preventive care, including conducting independent nursing consultations, cardiovascular risk assessment, lifestyle counseling, organization of screening programs, and inviting registered residents for preventive examinations. Therefore, nurses were purposively selected because they are the healthcare professionals most directly involved in cardiovascular prevention and screening activities within PHC (13).

Data collection continued until thematic saturation was reached, defined as the point at which no new codes or themes emerged from successive interviews. Similar responses were consistently observed after the first few interviews, and the final interviews did not generate additional themes. Interviews were conducted face-to-face using a paper-based interview guide. Instead of audio recording, detailed written notes were recorded during and immediately after each interview. This approach was chosen because the interviews were designed to capture participants' professional perspectives within the routine PHC setting. The interview guide included three core questions:

To what extent do you believe the collected cardiovascular health data reflect your practical experience? Are risk factors high among young adults, and what factors contribute to this?

How do cardiovascular risk factors differ between males and females, and what might explain this difference?

What interventions do you believe are needed to improve cardiovascular health among young people, particularly regarding CVD risk factors?

The interviews were conducted by researchers experienced in primary health care research. To minimize interpretation bias, three authors independently reviewed and manually coded the interview notes using conventional content analysis. Codes and emerging themes were compared, discussed, and refined until consensus was achieved. The qualitative component is reported in accordance with the Consolidated Criteria for Reporting Qualitative Research (COREQ). The completed COREQ checklist is provided as Supplementary material 2.

### Ethical approval

The study was approved by the Local Ethics Committee of the Khoja Ahmet Yassawi International Kazakh Turkish University, Turkistan, Kazakhstan (No.87, 14-11-2024), and conducted in accordance with the Declaration of Helsinki. All participants provided informed consent, acknowledged that their data would remain anonymous, and were informed of their right to withdraw from the study at any time.

## Results

### Overall sample characteristics

A total of 843 young adults participated in the study, including 432 urban (51.2%) and 411 rural (48.8%) residents. Overall, 289 participants (34.3%) were male and 554 (65.7%) were female. Most participants demonstrated intermediate cardiovascular health according to the LE8 (67.6%), whereas 28.9% achieved high cardiovascular health and 3.4% were classified as having poor cardiovascular health (Table 1).

**Table 1:** Distribution of lifestyle behaviors and cardiovascular health indicators by regions.

Variables		Urban	Rural	Total	*P*
		*N* (%)	*N* (%)	*N* (%)	
DASH group	Low commitment	212 (49.1%)	95 (23.1%)	307 (36.4%)	0.001
	Moderate commitment	216 (50.0%)	295 (71.8%)	511 (60.6%)	
	High commitment	4 (0.9%)	21 (5.1%)	25 (3.0%)	
	Total	432 (100.0)	411 (100.0)	843 (100.0)	
Physical activity	>150 min	298 (69.0)	388 (94.4)	686 (81.4)	0.001
	120–149	54 (12.5)	7 (1.7)	61 (7.2)	
	90–119	16 (3.7)	7 (1.7)	23 (2.7)	
	60–89	30 (6.9)	9 (2.2)	39 (4.6)	
	< 60	34 (7.9)	0 (0.0)	34 (4.0)	
Smoking group	Never smoker	162 (37.5)	37 (9.0)	199 (23.6)	0.001
	Former smoker. quit ≥5 y	216 (50.0)	35 (8.5)	251 (29.8)	
	Current smoker	54 (12.5)	339 (82.5)	393 (46.6)	
Sleep group	4–5 h	20 (4.6)	0 (0.0)	20 (2.4)	0.021
	< 4 hours	4 (0.9)	7 (1.7)	11 (1.3)	
	5–7 h	138 (31.9)	119 (29.0)	257 (30.5)	
	7–9 h	270 (62.5)	285 (69.3)	555 (65.8)	
Total Cholesterol	≥220	34 (7.9)	32 (7.8)	66 (7.8)	0.974
	190–219	6 (1.4)	6 (1.5)	12 (1.4)	
	160–189	38 (8.8)	38 (9.2)	76 (9.0)	
	130–159	93 (21.5)	84 (20.4)	177 (21.0)	
	< 130	261 (60.4)	251 (61.1)	512 (60.7)	
Blood Sugar	HbA_1_C ≥10.0 %	44 (10.2)	42 (10.2)	86 (10.2)	0.218
	HbA_1_C 7.0%−7.9 %	22 (5.1)	18 (4.4)	40 (4.7)	
	HbA_1_C < 7.0 % with diabetes	48 (11.1)	44 (10.7)	92 (10.9)	
	Fasting blood glucose 100–125 mg/dl or HbA_1_C 5.7%−6.5 % with no history of diabetes mellitus	104 (24.1)	96 (23.4)	200 (23.7)	
	Fasting blood glucose < 100 mg/dl or HbA_1_C < 5.7 % with no history of diabetes mellitus	214 (49.5)	211 (51.3)	425 (50.4)	
Blood pressure	Systolic BP ≥160 mmHg or Diastolic BP ≥100 mmHg	26 (6.0)	26 (6.3)	52 (6.2)	0.056
	Systolic BP 140–159 mmHg or Diastolic BP 90–99 mmHg	14 (3.2)	14 (3.4)	28 (3.3)	
	Systolic BP 130–139 mmHg or Diastolic BP 80–89 mmHg	34 (7.9)	32 (7.8)	66 (7.8)	
	Systolic BP 120–129 mmHg and Diastolic BP < 80 mmHg	102 (23.6)	98 (23.8)	200 (23.7)	
	Systolic BP < 120 mmHg and Diastolic BP < 80 mmHg	256 (59.3)	241 (58.6)	497 (59.0)	
Body Mass Index	>35	2 (0.5%)	28 (6.8%)	30 (3.6%)	0.001
	30–35	40 (9.3%)	40 (9.7%)	80 (9.5%)	
	25–29.9	168 (38.9%)	96 (23.4%)	264 (31.3%)	
	< 25	222 (51.4%)	247 (60.1%)	469 (55.6%)	
Gender	Male	220 (50.9)	69 (16.8)	289 (34.3)	0.001
	Female	212 (49.1)	342 (83.2)	554 (65.7)	
	Total	432 (100.0)	411 (100.0)	843 (100.0)	
Genetic factor (presence of heart disease or stroke in close relatives)	Yes	72 (16.7)	50 (12.2)	122 (14.5)	0.001
	No	298 (69.0)	344 (83.7)	642 (76.2)	
	Do not know	62 (14.4)	17 (4.1)	79 (9.4)	
Life's Essential 8	Low CVH	16 (3.7%)	13 (3.2%)	29 (3.4%)	0.001
	Moderate CVH	254 (58.8%)	316 (76.9%)	570 (67.6%)	
	High CVH	162 (37.5%)	82 (20.0%)	244 (28.9%)		

### Urban–rural differences in cardiovascular health

Clear urban–rural contrasts were observed across behavioral and clinical domains. Rural participants demonstrated healthier lifestyle behaviors, including greater adherence to the DASH dietary pattern (71.8 vs. 50.0% with moderate adherence and 5.1 vs. 0.9% with high adherence; *P* = 0.001) and markedly greater physical activity (94.4 vs. 69.0% achieving >150 min/week; *P* = 0.001). They also reported slightly better sleep duration (69.3 vs. 62.5%; *P* = 0.021). In contrast, smoking patterns diverged sharply, with rural residents showing extremely high rates of current smoking (82.5%), whereas urban participants were more often never or former smokers (*P* = 0.001). Despite these behavioral differences, clinical risk factors were largely similar between groups. No significant urban–rural differences were observed in blood lipids, glucose, or blood pressure (all *P* > 0.05). However, BMI distribution differed significantly between groups (*P* = 0.001), with rural participants showing a higher prevalence of obesity (BMI ≥30 kg/m^2^), although normal BMI (< 25 kg/m^2^) remained the most common category in both groups. Overall cardiovascular health also differed significantly between regions (*P* = 0.001). Urban participants more frequently achieved high cardiovascular health (37.5 vs. 20.0%), whereas rural participants were predominantly classified as having intermediate cardiovascular health (76.9 vs. 58.8%). The prevalence of poor cardiovascular health was low in both groups (3.7 vs. 3.2%) (Table 1).

### Life's essential 8 component scores by region

Consistent with categorical findings, rural participants scored higher in physical activity, diet, and sleep (all *P* ≤ 0.047), whereas urban participants had significantly better smoking scores (*P* < 0.001), reflecting lower tobacco exposure. Urban participants were also older on average (33.82 ± 9.69 vs. 30.85 ± 11.13 years; *P* < 0.001), which may partially influence risk profiles. No differences were observed in BMI, total cholesterol, HbA1c, or blood pressure. The overall LE8 score was significantly higher among urban participants than rural participants (74.52 ± 12.45 vs. 70.92 ± 10.48; P < 0.001), coinciding with higher smoking scores (Table 2).

**Table 2:** Comparison of life's essential 8 component scores by region among young adults (18–44 Years).

Variables	Gender			*P*
	Urban ($\bar{\text{x}}\pm \text{S}$)	Rural $\bar{\text{x}}\pm \text{S}$	Total $\bar{\text{x}}\pm \text{S}$	
Age	33.82 ± 9.69	30.85 ± 11.13	32.37 ± 10.52	< 0.001
Smoking	62.5 ± 33.11	13.26 ± 30.65	38.49 ± 40.31	< 0.001
Sleep	88.77 ± 16.81	91.06 ± 16.49	89.89 ± 16.69	0.047
Activity	86.39 ± 21.97	97.66 ± 10.21	91.89 ± 18.15	< 0.001
DASH	48.29 ± 14.82	56.44 ± 13.12	52.26 ± 14.59	< 0.001
Body mass index	81.39 ± 22.47	79.37 ± 30.74	80.4 ± 26.82	0.274
Total cholesterol (mg/dl)	77.13 ± 31.88	77.32 ± 31.94	77.22 ± 31.89	0.930
Current HbA1c (%)	69.95 ± 33.96	70.95 ± 33.98	70.44 ± 33.95	0.671
Blood pressure	81.71 ± 28.21	81.27 ± 28.62	81.49 ± 28.39	0.819
Life's essential 8 score	74.52 ± 12.45	70.92 ± 10.48	72.76 ± 11.67	< 0.001

### Gender differences in lifestyle behaviors by region

Urban–rural contrasts were also evident within gender analyses. In urban areas, smoking status (*P* < 0.001), blood pressure categories (*P* = 0.008), and overall cardiovascular health classification (*P* = 0.005) differed significantly between males and females. Urban females demonstrated higher proportions of high cardiovascular health (41.5 vs. 33.6%) and lower proportions of poor cardiovascular health (0.9 vs. 6.4%) than urban males. In rural areas, smoking remained the only lifestyle behavior that differed significantly between sexes (*P* = 0.015), whereas dietary adherence, physical activity, sleep, blood lipids, blood glucose, blood pressure, BMI, and overall cardiovascular health did not differ significantly (Table 3).

**Table 3:** Distribution of lifestyle behaviors and cardiovascular health indicators by regions.

Variables		Urban			*P* urban	Rural			*P* rural
		Male	Female	Total		Male	Female	Total	
		*N* (%)	*N* (%)	*N* (%)		*N* (%)	*N* (%)	*N* (%)	
DASH group	Low commitment	100 (45.5%)	112 (52.8%)	212 (49.1%)	0.304	18 (26.1%)	77 (22.5%)	95 (23.1%)	0.760
	Moderate commitment	118 (53.6%)	98 (46.2%)	216 (50.0%)		47 (68.1%)	248 (72.5%)	295 (71.8%)	
	High commitment	2 (0.9%)	2 (0.9%)	4 (0.9%)		4 (5.8%)	17 (5.0%)	21 (5.1%)	
	Total	220 (100.0)	212 (100.0)	432 (100.0)		69 (100.0)	342 (100.0)	411 (100.0)	
Physical activity	>150 min	104 (47.3)	110 (51.9)	214 (49.5)	0.211	65 (94.2)	323 (94.4)	388 (94.4)	0.817
	120–149	20 (9.1)	24 (11.3)	44 (10.2)		1 (1.4)	6 (1.8)	7 (1.7)	
	90–119	6 (2.7)	6 (2.8)	12 (2.8)		2 (2.9)	5 (1.5)	7 (1.7)	
	60–89	22 (10.0)	12 (5.7)	34 (7.9)		1 (1.4)	8 (2.3)	9 (2.2)	
	< 60	68 (30.9)	60 (28.3)	128 (29.6)		0 (0.0)	0 (0.0)	0 (0.0)	
Smoking group	Never smoker	54 (24.5)	108 (50.9)	162 (37.5)	< 0.001	5 (7.2)	32 (9.4)	37 (9.0)	0.015
	Former smoker. quit ≥5 y	134 (60.9)	82 (38.7)	216 (50.0)		–	35 (10.2)	35 (8.5)	
	Current smoker	32 (14.5)	22 (10.4)	54 (12.5)		64 (92.8)	275 (80.4)	339 (82.5)	
Sleep group	4–5 h	12 (5.5)	8 (3.8)	20 (4.6)	0.079	21 (30.4)	98 (28.7)	119 (29.0)	0.945
	< 4 h	4 (1.8)	0 (0.0)	4 (0.9)		1 (1.4)	6 (1.8)	7 (1.7)	
	5–7 h	76 (34.5)	62 (29.2)	138 (31.9)		21 (30.4)	98 (28.7)	119 (29.0)	
	7–9 h	128 (58.2)	142 (67.0)	270 (62.5)		47 (68.1)	238 (69.6)	285 (69.3)	
Total cholesterol	≥220	16 (7.3)	18 (8.5)	34 (7.9)	0.444	5 (7.2)	27 (7.9)	32 (7.8)	0.669
	190–219	2 (0.9)	4 (1.9)	6 (1.4)		2 (2.9)	4 (1.2)	6 (1.5)	
	160–189	24 (10.9)	14 (6.6)	38 (8.8)		4 (5.8)	34 (9.9)	38 (9.2)	
	130–159	44 (20.0)	49 (23.1)	93 (21.5)		15 (21.7)	69 (20.2)	84 (20.4)	
	< 130	134 (60.9)	127 (59.9)	261 (60.4)		43 (62.3)	208 (60.8)	251 (61.1)	
Blood Sugar	HbA_1_C_1_C ≥10.0 %	20 (9.1)	24 (11.3)	44 (10.2)	0.323	9 (13.0)	33 (9.6)	42 (10.2)	0.301
	HbA_1_C 7.0%−7.9%	8 (3.6)	14 (6.6)	22 (5.1)		–	18 (5.3)	18 (4.4)	
	HbA_1_C < 7.0% with diabetes	26 (11.8)	22 (10.4)	48 (11.1)		6 (8.7)	38 (11.1)	44 (10.7)	
	Fasting blood glucose 100–125 mg/dl or HbA_1_C 5.7%−6.5 % with no history of diabetes mellitus	60 (27.3)	44 (20.8)	104 (24.1)		18 (26.1)	78 (22.8)	96 (23.4)	
	Fasting blood glucose < 100 mg/dl or HbA_1_C < 5.7 % with no history of diabetes mellitus	106 (48.2)	108 (50.9)	214 (49.5)		36 (52.2)	175 (51.2)	211 (51.3)	
Blood pressure	Systolic BP ≥160 mmHg or Diastolic BP ≥100 mmHg	12 (5.5)	14 (6.6)	26 (6.0)	0.008	4 (5.8)	22 (6.4)	26 (6.3)	0.981
	Systolic BP 140–159 mmHg or Diastolic BP 90–99 mmHg	10 (4.5)	4 (1.9)	14 (3.2)		2 (2.9)	12 (3.5)	14 (3.4)	
	Systolic BP 130–139 mmHg or Diastolic BP 80–89 mmHg	24 (10.9)	10 (4.7)	34 (7.9)		6 (8.7)	26 (7.6)	32 (7.8)	
	Systolic BP 120–129 mmHg and Diastolic BP < 80 mmHg	40 (18.2)	62 (29.2)	102 (23.6)		15 (21.7)	83 (24.3)	98 (23.8)	
	Systolic BP < 120 mmHg and Diastolic BP < 80 mmHg	134 (60.9)	122 (57.5)	256 (59.3)		42 (60.9)	199 (58.2)	241 (58.6)	
Body Mass Index	>35	2 (0.9%)	–	2 (0.5%)	0.149	4 (5.8%)	24 (7.0%)	28 (6.8%)	0.504
	30–35	22 (10.0%)	18 (8.5%)	40 (9.3%)		6 (8.7%)	34 (9.9%)	40 (9.7%)	
	25–29.9	93 (42.3%)	75 (35.4%)	168 (38.9%)		21 (30.4%)	75 (21.9%)	96 (23.4%)	
	< 25	103 (46.8%)	119 (56.1%)	222 (51.4%)		38 (55.1%)	209 (61.1%)	247 (60.1%)	
Life's Essential 8	Low CVH	14 (6.4%)	2 (.9%)	16 (3.7%)	0.005	3 (4.3%)	10 (2.9%)	13 (3.2%)	0.811
	Moderate CVH	132 (60.0%)	122 (57.5%)	254 (58.8%)		53 (76.8%)	263 (76.9%)	316 (76.9%)	
	High CVH	74 (33.6%)	88 (41.5%)	162 (37.5%)		13 (18.8%)	69 (20.2%)	82 (20.0%)		

### Gender comparison of life's essential 8 scores by region

In urban participants, females had higher overall LE8 scores than males (74.41 ± 11.68 vs. 71.07 ± 14.00; *P* = 0.020), mainly due to better smoking and sleep scores. In contrast, rural populations showed no significant gender differences in overall or individual component scores, indicating a more uniform cardiovascular health profile across sexes (Table 4).

**Table 4:** Comparison of life's essential 8 component scores by gender among young adults (18–44 Years) in regions.

Variables	Urban			*P*	Rural			*P*
	Male ($\bar{\text{x}}\pm \text{S}$)	Female $\bar{\text{x}}\pm \text{S}$	Total $\bar{\text{x}}\pm \text{S}$		Male ($\bar{\text{x}}\pm \text{S}$)	Female $\bar{\text{x}}\pm \text{S}$	Total $\bar{\text{x}}\pm \text{S}$	
Age	35.56 ± 10.51	32.02 ± 8.41	33.82 ± 9.69	< 0.001	31.78 ± 11.11	30.66 ± 11.14	30.85 ± 11.13	0.447
Smoking score	55.00 ± 30.93	70.28 ± 33.57	62.5 ± 33.11	< 0.001	7.25 ± 26.12	14.47 ± 31.38	13.26 ± 30.65	0.074
Sleep score	86.82 ± 19.03	90.80 ± 13.91	88.77 ± 16.81	0.014	90.94 ± 16.03	91.08 ± 16.61	91.06 ± 16.49	0.949
Diet score	48.95 ± 15.29	47.59 ± 14.31	48.29 ± 14.82	0.341	57.61 ± 13.84	56.2 ± 12.98	56.44 ± 13.12	0.416
Physical activity	62.63 ± 42.64	67.37 ± 41.41	64.96 ± 42.06	0.242	97.83 ± 9.37	97.63 ± 10.39	97.66 ± 10.21	0.885
Body mass index	79.41 ± 23.23	83.44 ± 21.51	81.39 ± 22.47	0.062	78.99 ± 29.16	79.44 ± 31.09	79.37 ± 30.74	0.910
Total cholesterol score	77.45 ± 31.53	76.79 ± 32.31	77.13 ± 31.88	0.829	78.26 ± 31.76	77.13 ± 32.02	77.32 ± 31.94	0.790
Blood sugar score	70.36 ± 32.80	69.53 ± 35.19	69.95 ± 33.96	0.799	71.3 ± 35.06	70.88 ± 33.81	70.95 ± 33.98	0.924
Blood pressure score	81.14 ± 28.90	82.31 ± 27.53	81.71 ± 28.21	0.666	82.25 ± 28.15	81.07 ± 28.75	81.27 ± 28.62	0.755
Life's essential 8 score	71.07 ± 14.00	74.41 ± 11.68	72.71 ± 13.01	0.020	70.55 ± 10.88	70.99 ± 10.42	70.92 ± 10.48	0.753

Overall, rural participants demonstrated more favorable dietary patterns, higher physical activity, and longer sleep duration; however, they also had substantially higher smoking prevalence and obesity. Conversely, urban participants achieved higher overall LE8 scores, largely driven by markedly better smoking scores. These findings should be interpreted in the context of the substantial gender imbalance between the urban and rural samples.

### Qualitative findings

A total of seven nurses from five PHC participated in the interviews.

### Age and onset of risk factors

Most nurses reported that cardiovascular risk factors become more apparent around the age of 40, particularly obesity and early signs of hypertension.

“*We usually start seeing obesity and elevated blood pressure becoming more common after the age of 40.” (N 2)*

Two nurses noted that although awareness of risk factors and discussions around nutrition have increased over the past decade, cultural norms and lifestyle habits continue to shape behavior. One nurse suggested that economic stress and limited local employment opportunities may negatively influence health practices, indicating that future improvements in economic conditions could lead to better health attitudes. Access to healthy food was also described as being influenced by affordability, with fast food sometimes perceived as more accessible despite healthier options being available.

### Gender differences at risk

The majority nurses agreed that male individuals demonstrate higher cardiovascular risk than females. Male participants were described as having higher rates of obesity and smoking, particularly among those who are married, which nurses believed exacerbated their cardiovascular health. Three nurses emphasized that male patients rarely seek medical care, especially for preventive purposes, leading to delayed detection and management of risk factors.

“*Young males rarely come unless they already have symptoms.” (N 5)*.

### Need for stronger preventive measures

Participants stressed the need to strengthen cardiovascular prevention efforts and public health promotion, noting constraints such as limited staffing time and resources.

“*If screening were organized at workplaces, many more males would participate.” (N 6)* .

Nurses suggested reviving workplace-based health programs, where employers collaborate with clinics to provide educational sessions and basic screening directly in workplaces, potentially improving participation, particularly among male populations, who are less likely to visit PHC.

## Discussion

In this mixed -methods study of young adults aged 18–44 years in the Turkestan region, we found that most participants demonstrated moderate cardiovascular health (CVH) according to the LE8 construct. Approximately two-thirds of participants (67.6%) demonstrated intermediate CVH, whereas 28.9% achieved high and only 3.4% were classified as having poor CVH. However, several modifiable risk factors, particularly suboptimal diet, insufficient physical activity in nearly one-third of participants, excess weight, and impaired glucose regulation, remain areas of concern and may contribute to future CVD burden. The mean overall LE8 score in our population was higher than that reported among young adults in the U.S. National Health and Nutrition Examination Survey (NHANES), where the overall mean CVH score was 69.2 (14). Although the mean LE8 score in our study was higher than that reported in NHANES, these findings should be interpreted cautiously because the study populations differ substantially in age distribution, ethnicity, healthcare systems, and sociocultural context. Therefore, this comparison is intended only to provide general context rather than a direct comparison of CVH across populations. The observed difference may reflect contextual factors such as the younger median age of our participants and the availability of routine blood pressure and glucose screening within PHC settings.

The long-term behavioral consequences of the COVID-19 pandemic, including reduced physical activity, changes in dietary habits, and increased psychosocial stress, may also have affected cardiovascular health among young adults (15, 16). Although our study was not designed to evaluate these effects, they should be considered when interpreting the findings.

An important finding of this study was the presence of clear urban–rural differences in CVH profiles. Rural participants demonstrated healthier lifestyle behaviors overall, including better adherence to DASH dietary recommendations, substantially higher physical activity levels, and slightly better sleep duration compared with urban residents. These findings are consistent with evidence from other low- and middle-income countries, where rural populations often maintain more traditional dietary patterns and higher levels of occupational or transportation-related physical activity compared with increasingly sedentary urban populations undergoing rapid lifestyle transition (17). The coexistence of high physical activity and obesity may reflect the nutrition transition occurring in rural communities, where physically demanding occupations persist but dietary patterns have shifted toward increased caloric intake and processed foods.

Despite these favorable lifestyle patterns, rural participants also demonstrated markedly higher smoking prevalence and a greater burden of obesity. In contrast, urban participants had substantially lower tobacco exposure and achieved higher overall LE8 scores despite poorer diet and lower physical activity. This apparent paradox likely reflects the substantial weighting of smoking within the LE8 composite score, which offset the more favorable diet, physical activity, and sleep profiles observed among rural participants. Similar studies have shown that rural populations may experience a dual burden of protective traditional lifestyle behaviors alongside increasing obesity and persistent tobacco exposure (18). The extremely high smoking prevalence observed in rural populations is particularly concerning and suggests that tobacco use remains strongly embedded within local sociocultural norms (19).

Interestingly, despite behavioral differences between urban and rural groups, no significant differences were observed in blood pressure, HbA1c, lipid levels, or most clinical indicators. This may reflect the relatively young age of participants, where adverse lifestyle behaviors have not yet translated into clinically detectable cardiometabolic disease. However, the coexistence of obesity and heavy smoking among rural participants raises concern for a potentially substantial future increase in CVD burden as this population ages. These findings reinforce the importance of early prevention strategies targeting young adults before clinical disease becomes established.

Gender differences observed in this study were consistent with international literature. Females demonstrated more favorable CVH profiles, particularly in urban settings, where they showed lower smoking exposure, better sleep, and higher overall LE8 scores than males. Similar findings have been reported in international cohorts, where females generally demonstrate healthier cardiovascular behaviors and better overall CVH metrics (19). Such differences may partially explain the shorter life expectancy among males in Kazakhstan and the persistently high burden of preventable cardiovascular mortality. Recent studies from Kazakhstan have demonstrated continuing high rates of avoidable mortality and cardiovascular disease mortality despite ongoing investments in cardiovascular treatment and care systems (11, 20). These findings suggest that behavioral risk factors and insufficient engagement in preventive care remain major contributors to premature mortality among males.

The marked predominance of females in the rural sample should also be considered when interpreting the urban–rural comparisons. Although participants were recruited from the registered PHC population through preventive invitations, participation required attendance at the PHC facility. Young males, particularly in rural areas, may have been less likely to participate in preventive examinations because of work-related commitments and lower engagement with preventive health services. Consequently, some of the observed urban–rural differences may partly reflect differential participation by sex rather than true population differences.

Urban–rural gender analyses further highlighted important contextual differences. In urban populations, females demonstrated more favorable cardiovascular health profiles, particularly with respect to smoking, sleep quality, and overall LE8 scores. In contrast, rural males and females exhibited largely similar cardiovascular health profiles, with smoking remaining the only behavior that differed significantly between sexes. This finding may indicate stronger normalization of tobacco use within rural communities and suggests that rural females may not experience the same protective behavioral patterns observed among urban females.

Our qualitative findings provided important contextual interpretation of the quantitative results. Nurses reported that cardiovascular risk factors become increasingly apparent around the age of 40 years, especially obesity and early hypertension. Participants also emphasized the role of cultural dietary habits, economic stress, and affordability of unhealthy foods in shaping lifestyle behaviors. These findings may help explain the coexistence of high physical activity and obesity observed among rural participants, where dietary quality and caloric intake may offset some protective effects of physical activity.

Another important qualitative finding was the consistently low engagement of males in preventive health care. Nurses described males as less likely to attend PHC facilities unless symptoms become severe, resulting in delayed identification and management of cardiovascular risk factors. Similar barriers have been described internationally, including medical mistrust, masculinity-related norms, work-related constraints, and low perceived need for preventive care (21, 22). Participants therefore recommended strengthening workplace-based and community-based prevention programs, where screening and health education could be delivered directly within workplaces and local communities. Such strategies may improve participation among young adults, particularly males and rural residents who may otherwise have limited engagement with PHC services.

Although Kazakhstan has expanded access to advanced cardiovascular interventions and specialized cardiac care in recent years, prevention of modifiable cardiovascular risk factors remains a major public health priority (8, 23). Regional and cultural differences may also influence cardiovascular health patterns within the country. Previous studies have demonstrated ethnic and regional differences in mortality patterns across Kazakhstan, likely reflecting variations in socioeconomic conditions, lifestyle behaviors, and cultural norms (24). Therefore, cardiovascular prevention strategies should not rely on uniform national approaches alone but should instead account for important urban–rural and gender-specific differences in cardiovascular health behaviors. Rural populations may particularly benefit from intensive tobacco-control initiatives and obesity prevention programs, whereas urban interventions should prioritize improving physical activity and dietary quality. Strengthening preventive services within PHC, expanding workplace screening initiatives, and implementing culturally tailored health promotion strategies may help reduce future cardiovascular disease burden among young adults in Kazakhstan.

### Strengths and limitations

A major strength of this study is that it represents the first investigation in Kazakhstan to assess cardiovascular health among young adults using the LE8 framework, providing important baseline evidence for a population that has been largely overlooked in previous national research, which has traditionally focused on older adults. Another strength is the mixed-methods design, which not only quantified cardiovascular health status but also incorporated the perspectives of PHC nurses. This allowed contextual interpretation of behavioral and health system determinants and provided practical insights into the implementation of preventive strategies within PHC settings.

However, several limitations should be acknowledged. Although participants were recruited from the registered PHC population through preventive invitations rather than exclusively among patients seeking medical care, participation still required attendance at the PHC facility. Consequently, individuals who were less likely to participate in preventive examinations, particularly young males, may have been underrepresented. This may have contributed to the predominance of women in the rural sample and influenced the magnitude of the observed gender and urban–rural differences. Therefore, the findings should be interpreted as representative of young adults who participated in PHC-based preventive assessment rather than the entire young adult population of the Turkestan region. Second, although clinical indicators such as blood pressure, glucose, and lipid levels were objectively measured, several LE 8 components relied on self-reported behaviors and may therefore be subject to recall or social desirability bias. Third, the cross-sectional design precludes assessment of causal relationships between cardiovascular health status and future disease outcomes. The qualitative component included seven PHC nurses from five PHC centers. However, nurses were purposively selected because they are primarily responsible for preventive examinations and screening programs within the Kazakhstan PHC system. Although thematic saturation was achieved, the findings may not fully reflect the perspectives of physicians or other healthcare professionals involved in cardiovascular prevention. In addition, interviews were documented using written notes rather than audio recordings, member checking was not performed, and formal inter-rater reliability statistics were not calculated. Coding consistency was established through independent coding by three researchers followed by discussion and consensus.

### Implications for future research and public health policy

Future research should expand this work across multiple regions of Kazakhstan, as regional variation in cultural norms, lifestyle behaviors, and alcohol consumption may substantially influence cardiovascular health patterns. Previous studies have reported higher alcohol consumption in northern regions of Kazakhstan, partly attributed to sociocultural influences among populations of Russian origin, which may differ considerably from the southern region where this study was conducted (24). Therefore, assessment of cardiovascular health across regions is important to better understand geographic disparities and to develop culturally tailored prevention strategies. Future studies should also aim to recruit nationally representative samples with balanced participation of men and women and apply standardized Life's Essential 8 assessment to improve comparability across regions and over time. In addition, longitudinal studies are needed to determine whether young adults with low or moderate cardiovascular health progress to early cardiovascular disease and to evaluate the long-term effectiveness of workplace-based and PHC-led prevention programs. From a public health perspective, our findings support strengthening primary health care–based prevention, particularly through targeted tobacco-control measures, workplace and community screening programs, and region- and gender-specific interventions for young adults.

## Conclusion

This study demonstrated that most young adults in the Turkestan region had moderate cardiovascular health according to the LE8 framework; however, several modifiable cardiovascular risk factors remain highly prevalent. Important urban–rural differences were identified. Rural participants demonstrated healthier lifestyle behaviors, including better diet quality, higher physical activity, and longer sleep duration, but also had substantially higher smoking prevalence and greater obesity burden. In contrast, urban participants achieved higher overall LE8 scores, primarily due to lower tobacco exposure despite less favorable diet and physical activity patterns. Gender disparities were also evident, with females generally demonstrating healthier cardiovascular profiles than males, particularly in urban settings. Qualitative findings further highlighted the influence of cultural norms, economic stress, unhealthy dietary practices, and low engagement of men in preventive health care. Nurses emphasized the need for stronger workplace-based and community-based prevention programs to improve early identification and management of cardiovascular risk factors. These findings highlight the need for region-specific and gender-sensitive cardiovascular prevention strategies within primary health care. Strengthening tobacco-control measures, expanding workplace- and community-based prevention programs, and improving engagement of young men in preventive services may help reduce the future burden of cardiovascular disease among young adults in Kazakhstan.

## Data Availability

The raw data supporting the conclusions of this article will be made available by the authors, without undue reservation.
