# Supplementary material for: Urban–rural differences in cardiovascular health among young adults in the Turkestan region of Kazakhstan
Source: Front Public Health. 2026 Aug 19;14:1873662. doi: 10.3389/fpubh.2026.1873662 (PMC13534027; doi:10.3389/fpubh.2026.1873662)
Supplement: Supplementary file 1 [file Supplementary_file_1.docx]

Supplementary Material 1. Participant characteristics

| Participant ID | Sex | Age (years) | Professional experience (years) | PHC location | Position/Role |
| --- | --- | --- | --- | --- | --- |
| N1 | Female | 42 | 18 | Urban | PHC nurse responsible for screening and preventive programmes |
| N2 | Female | 38 | 14 | Urban | PHC nurse responsible for screening and preventive programmes |
| N3 | Female | 45 | 20 | Rural | PHC nurse responsible for screening and preventive programmes |
| N4 | Female | 36 | 11 | Rural | PHC nurse responsible for screening and preventive programmes |
| N5 | Female | 40 | 16 | Rural | PHC nurse responsible for screening and preventive programmes |
| N6 | Female | 34 | 9 | Urban | PHC nurse responsible for screening and preventive programmes |
| N7 | Female | 47 | 23 | Rural | PHC nurse responsible for screening and preventive programmes |

Supplementary Material 2. COREQ (Consolidated Criteria for Reporting Qualitative Research) Checklist

| Item | Guide question | Reported in manuscript |
| --- | --- | --- |
| Domain 1: Research team and reflexivity | | |
| 1 | Interviewer/facilitator | Interviews were conducted by members of the research team experienced in primary health care research. |
| 2 | Credentials | Researchers with experience in public health and primary health care research. |
| 3 | Occupation | Academic researchers. |
| 4 | Gender | Not reported. |
| 5 | Experience and training | Interviewers had previous experience conducting qualitative research in PHC settings. |
| 6 | Relationship established | No prior relationship with participants was established before the interviews. |
| 7 | Participant knowledge of the interviewer | Participants were informed about the purpose of the study and the researchers' roles before providing consent. |
| 8 | Interviewer characteristics | Researchers aimed to minimize interpretation bias through independent coding and consensus discussions. |
| Domain 2: Study design | | |
| 9 | Methodological orientation | Qualitative descriptive design using conventional content analysis. |
| 10 | Sampling | Purposive sampling of PHC nurses responsible for preventive care and screening programmes. |
| 11 | Method of approach | Face-to-face invitation. |
| 12 | Sample size | Seven nurses from five PHC centres. |
| 13 | Non-participation | No eligible participants declined participation. *(If true; otherwise report actual number.)* |
| 14 | Setting | Primary health care centres in the Turkestan region. Kazakhstan. |
| 15 | Presence of non-participants | Only interviewer and participant were present. *(If true.)* |
| 16 | Description of sample | Nurses responsible for cardiovascular prevention and national screening programmes according to the Kazakhstan PHC Standard. |
| 17 | Interview guide | Semi-structured interview guide with three predefined open-ended questions. |
| 18 | Repeat interviews | No repeat interviews were conducted. |
| 19 | Audio/visual recording | No audio recording. Detailed written notes were taken during and immediately after interviews. |
| 20 | Field notes | Yes. Detailed field notes were recorded. |
| 21 | Duration | Approximately 20–30 minutes per interview. *(Replace with your actual duration.)* |
| 22 | Data saturation | Data collection continued until no new codes or themes emerged from successive interviews. |
| 23 | Transcripts returned | No member checking was performed. |
| Domain 3: Analysis and findings | | |
| 24 | Number of data coders | Three authors independently coded the interview notes. |
| 25 | Description of coding tree | No formal coding tree presented. |
| 26 | Derivation of themes | Themes were derived inductively using conventional content analysis. |
| 27 | Software | Manual coding; no qualitative software was used. |
| 28 | Participant checking | Not performed. |
| 29 | Quotations presented | Yes. Representative quotes are presented to illustrate the major themes. |
| 30 | Data and findings consistent | Yes. Themes are supported by interview summaries. |
| 31 | Clarity of major themes | Yes. Major themes are presented under separate headings. |
| 32 | Clarity of minor themes | Minor viewpoints are described where appropriate. |
